# Absolute dosimetry for FLASH proton pencil beam scanning radiotherapy

**DOI:** 10.1038/s41598-023-28192-0

**Published:** 2023-02-04

**Authors:** Ana Lourenço, Anna Subiel, Nigel Lee, Sam Flynn, John Cotterill, David Shipley, Francesco Romano, Joe Speth, Eunsin Lee, Yongbin Zhang, Zhiyan Xiao, Anthony Mascia, Richard A. Amos, Hugo Palmans, Russell Thomas

**Affiliations:** 1 National Physical Laboratory, Teddington, TW11 0LW UK; 2 University College London, London, WC1E 6BT UK; 3 University of Birmingham, Birmingham, B15 2TT UK; 4 Istituto Nazionale di Fisica Nucleare, Sezione di Catania, Via S Sofia 64, 95123 Catania, Italy; 5 Cincinnati Children’s Hospital Medical Center, Cincinnati, OH USA; 6 University of Cincinnati Medical Center, Cincinnati, OH USA; 7 MedAustron Ion Therapy Center, A-2700 Wiener Neustadt, Austria; 8 University of Surrey, Guildford, GU2 7XH UK

**Keywords:** Applied physics, Electronics, photonics and device physics

## Abstract

A paradigm shift is occurring in clinical oncology exploiting the recent discovery that short pulses of ultra-high dose rate (UHDR) radiation—FLASH radiotherapy—can significantly spare healthy tissues whilst still being at least as effective in curing cancer as radiotherapy at conventional dose rates. These properties promise reduced post-treatment complications, whilst improving patient access to proton beam radiotherapy and reducing costs. However, accurate dosimetry at UHDR is extremely complicated. This work presents measurements performed with a primary-standard proton calorimeter and derivation of the required correction factors needed to determine absolute dose for FLASH proton beam radiotherapy with an uncertainty of 0.9% (1$\sigma$), in line with that of conventional treatments. The establishment of a primary standard for FLASH proton radiotherapy improves accuracy and consistency of the dose delivered and is crucial for the safe implementation of clinical trials, and beyond, for this new treatment modality.

## Introduction

Radiotherapy, alone or in combination with other cancer therapies, is used as a treatment regimen in approximately 50% of cancer cases and is by far the most cost-effective method of cancer treatment^1^. The development of technologically advanced methods of targeted delivery of radiation to tumours, including intensity-modulated radiotherapy, image-guided radiotherapy, stereotactic radiosurgery, and the introduction of proton beam radiotherapy has led to improvements in patient survival^2–4^. However, during any radiotherapy treatment, apart from the targeted delivery of radiation to the tumour, some undesirable but unavoidable dose will also be deposited to the normal healthy tissues surrounding the targeted tumour. For the last century, radiotherapy has predominately been delivered in fractionated doses over a period of several weeks, typically 30 separate fractions, to allow time for the irradiated normal tissues to recover between each treatment and to help reduce the effects of radiation toxicity. Nevertheless, these normal tissue toxicities are a limiting factor for curative radiotherapy outcomes^5–9^. Moreover, improved cancer survival amplifies the negative impact of late toxicities resulting from radiotherapy treatment, subsequently having a detrimental impact on the quality of life of cancer survivors. This drives further efforts to continue technological developments which boost the efficacy of radiotherapy treatments and increase the therapeutic window by enhancing tumour control probability (TCP) and lowering normal tissue complication probability (NTCP).

Recent studies with ultra-high dose rates (UHDR) (averaged dose rate > 40 Gy s^−1^), known as FLASH radiotherapy, have demonstrated a remarkable reduction in normal tissue toxicity with respect to conventional dose-rate radiotherapy (0.1–1 Gy s^−1^) while maintaining similar tumour response^10–14^. In 2019, the first patient was treated with FLASH radiotherapy employing a 5.6 MeV electron beam^15^. More recently, the first FLASH proton beam radiotherapy clinical trial commenced at the Cincinnati Children’s Hospital Medical Center (CCHMC), USA, to treat patients with symptomatic metastatic bone cancer^16^. The properties of FLASH proton beam radiotherapy promise considerable reduction in post-treatment complications whilst improving patient access and reducing the cost of proton beam radiotherapy because of significant increase of patient throughput. This is due to the considerably shorter treatment times and the potential for very few, even single, treatment fractions, whilst in equivalent conventional dose-rate radiotherapy treatments, 30 fractions would be delivered over approximately 6 weeks.

Accurate dosimetry is crucial for the safe implementation of any radiotherapy technique. It ensures best practice and consistency of treatments across radiotherapy centres and modalities, both nationally and internationally. In the protocols for dosimetry of proton beam radiotherapy^17,18^, reference dosimetry is based on ionisation chamber measurements traceable to calibrations made in ^60^Co beams. Although these protocols are accepted for current clinical practice, they do not include recommendations for treatments using UHDR. Ionisation chambers exhibit significant ion recombination effects^19–24^ in UHDR beams requiring a large correction, burdened with substantial uncertainty. Dosimetry at UHDR is complicated and it is essential to understand the effects that impact detector response in this radiotherapy modality. An extensive review on detectors used in UHDR beams can be found in^25^. The use of a Faraday cup for the commission of a beam monitor at UHDR has been reported^26^. Passive detectors such as alanine^27^, radiochromic films, and thermoluminescent dosimeters (TLDs) have also been used^19,28^. Apart from careful calibration, these detectors also require time-consuming post-irradiation processing, and their behaviour has not yet been well-tested and documented for routine use with UHDR irradiations. Therefore, it is essential to establish a good understanding of the fundamental dosimetric challenges in UHDR beams to avoid dosimetric errors which may lead to both the incorrect quantification of dose delivered to the patient and flawed interpretation of the clinical outcomes related to the FLASH effect. Moreover, lack of good practice guidelines and dosimetric protocols for this new radiotherapy modality necessitate an independent verification of the beam output by employing absolute dosimetry based on a primary standard measurement, especially for clinical implementation. Absolute dosimetry refers to the direct measurement of dose from fundamental principles and realised by an absorbed dose primary-standard measurement standard, which can provide independent dosimetric validation for the treatment centre or hospital implementing FLASH radiotherapy. Calorimetry is the only direct method to measure the quantity of interest in radiotherapy^29^, which is absorbed dose, and its feasibility in conventional proton beams has been demonstrated^30,31^. Note that absorbed dose-to-water is the relevant quantity, as opposed to dose-to-tissue, because almost all radiation damage of the cell is initiated by the radiolysis of water. In calorimetry, the energy absorbed is determined by measurement of the radiation-induced temperature rise in combination with accurate knowledge of the specific heat capacity of the medium and beam-dependent correction factors. In this work, pioneering measurements were performed using a primary-standard proton calorimeter (PSPC) developed at the National Physical Laboratory (NPL), the UK’s National Measurement Institute, and the necessary corrections calculated to enable derivation of absolute dose for FLASH proton beam radiotherapy for the implementation of the first in-human clinical trial at CCHMC using this new modality. Ionisation chamber measurements were also performed, following recommendations of the accepted protocols^17,18^ for dosimetry of clinical proton beams, to test their suitability and limitations at UHDR by benchmarking their response against the NPL PSPC.

## Results and discussion

The experiments were carried out at the 250 MeV clinical proton beamline at the CCHMC, where protons are accelerated by a cyclotron. Measurements were performed in six rectangular fields developed for the treatment of symptomatic bone metastasis with an averaged dose rate of ~ 63 Gy s^−1^^32^, using a 250 MeV mono-energetic scanned layer, which corresponds to a depth of penetration in water of 38 g cm^−2^. For all fields, the physical dose delivered was of the order of 8 Gy, according to the requirements of the FAST-01 trial.

### NPL PSPC

**Figure 1:**
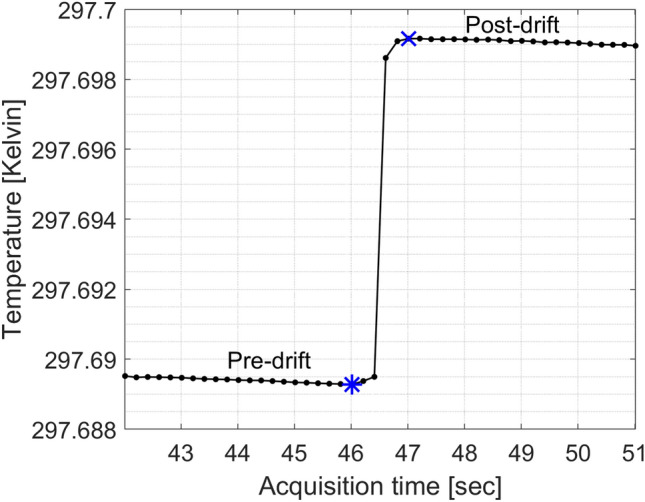
Measured temperature in Kelvin using the NPL PSPC for a FLASH irradiation. The symbols in blue mark approximately the start (*) and end (x) of irradiation as well as the end and start of the pre- and post- irradiation temperature drift curves, respectively.

The NPL PSPC is a graphite calorimeter that can measure changes in temperature very accurately (with an uncertainty better than 100 µK) by thermistors embedded in each of the calorimeter graphite components. Figure 1 shows a calorimeter run acquired at CCHMC for a FLASH irradiation. The change in temperature (of the level of 10 mK) is clearly visible between the time points when the irradiation starts and ends. The absolute measured temperature of the core is of the order of 297.7 K. This is because the outer jacket is kept at a controlled temperature (~ 298.2 K), elevated above the room temperature, to minimise the effect of changes in room temperature impacting the temperature of the core.

**Figure 2:**
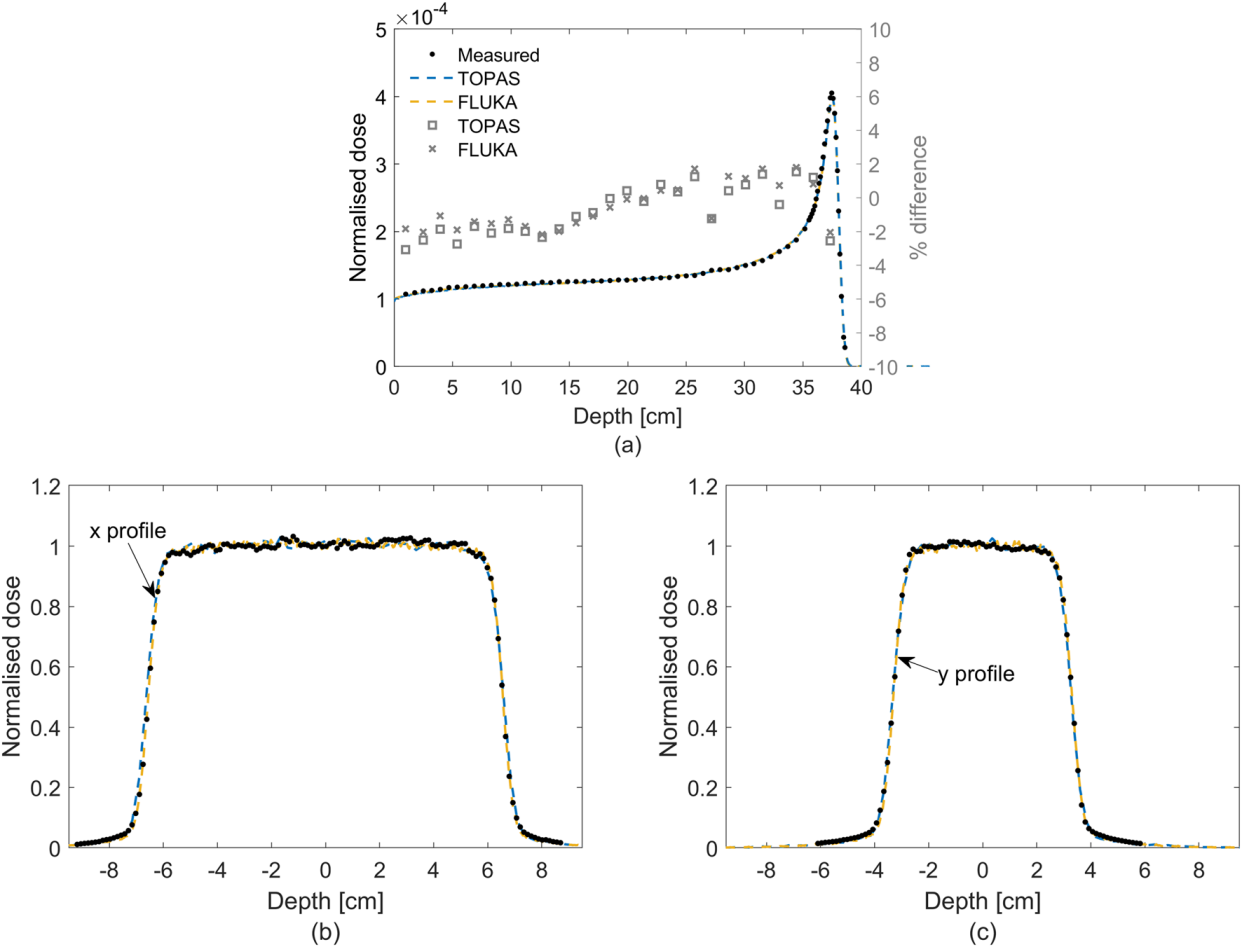
Measured and simulated dose distributions for the field 12 × 5 cm^2^. Depth- (**a**) and lateral-dose distributions (**b**,**c**). Solid circles represent the measured data, dashed curves represent the simulated data, and open squares and crosses the percentage difference between simulated and measured data using TOPAS and FLUKA, respectively. The field sizes were defined at the 90% of the isodose line. For simplification, the fields were named to the field size rounded down to the nearest integer.

To determine the Monte Carlo derived corrections for the NPL PSPC, accurate modelling of the beam parameters is essential. Figure 2 shows the laterally integrated depth-dose (IDD) curve obtained from a large-area ionisation chamber (IBA StingRay) as well as the lateral profiles measured using Gafchromic® film (EBT3, Ashland, NJ), using a 250 MeV mono-energetic beam for one of the fields tested. Data from Monte Carlo simulations are also presented after the tuning of the simulated beam parameters, showing good agreement between measured and simulated depth- and lateral-dose distributions. The IDDs were normalised to the area under the curves and the lateral profiles were normalised to the averaged dose delivered to avoid systematic errors. The optimal beam parameters found were as follows. For TOPAS the beam energy was tuned to 249.7 MeV, energy spread of σ = 0.2 MeV and divergency of σ = 3.5 mrad, and for FLUKA the beam energy was tuned to 249.3 MeV, energy spread of σ = 0.0 MeV and divergency of σ = 3.3 mrad. The quantities determined for the NPL PSPC used to measure the FLASH proton beam at CCHMC are listed in Table 1. The quantities comprise the specific heat capacity of graphite, $c$, and the various beam-dependent correction factors applied: an impurity correction factor, ${k}_{\mathrm{imp}}$ (to account for the presence of thermistors, epoxy and thermistor wires in the calorimeter core), a gap correction factor, ${k}_{\mathrm{gap}}$ (accounting for the presence of vacuum gaps that minimise heat transfers to and from the environment), a dose conversion factor, ${s}_{\mathrm{w},\mathrm{g}}\cdot {k}_{\mathrm{fl}}$ (defined as the product of the water-to-graphite mass-stopping-power ratio and the fluence correction factor, which convert the quantity measured, dose-to-graphite to the quantity of interest, dose-to-water) and ${k}_{\mathrm{z},\; {\mathrm{cal}}}$ (corrects for the reference distance from the beam’s source). Note that $c$ was measured experimentally, ${k}_{\mathrm{imp}}$, ${k}_{\mathrm{gap}}$, ${s}_{\mathrm{w},\mathrm{g}}$ and ${k}_{\mathrm{fl}}$ were determined numerically using Monte Carlo simulations and ${k}_{\mathrm{z},\mathrm{ cal}}$ was determined analytically (more details about the calorimeter corrections are given in the methods section). The correction factors ${k}_{\mathrm{imp}}$, ${k}_{\mathrm{gap}}$ and ${k}_{\mathrm{z},\mathrm{ cal}}$ were close to unity whereas ${s}_{\mathrm{w},\mathrm{g}}$ and ${k}_{\mathrm{fl}}$ amounted to 1.1210 and 0.9713, respectively. The value found for the ${s}_{\mathrm{w},\mathrm{g}}$ is consistent with previously reported values^31,33^. The ${k}_{\mathrm{fl}}$ factor accounts for the difference in fluence between water and graphite at water-equivalent depths and results from the difference between non-elastic nuclear interaction cross-sections as well as the production cross sections for secondary particles for those materials. The particles that contribute the most to the fluence correction factor between water and graphite are protons and alpha particles. In an irradiated field of 12 × 5 cm^2^, the difference in proton fluence is mainly due to the lack of lateral charged particle equilibrium within the area of the calorimeter core, which has a radius of 0.8 cm. For a 250 MeV proton beam, the range of secondary protons can be up to 13 cm. Water contains free hydrogen, which undergoes a head-on elastic collision with the incident protons and produces secondary protons with larger scattered angles in comparison with graphite, hence escaping the area of the calorimeter core and reducing the ${k}_{\mathrm{fl}}$ correction factor. Moreover, the production cross section of alpha particles per unit of atomic weight of the target atom is 6% larger for incident protons of 250 MeV impinging on a carbon atom than on an oxygen atom^34^.

**Table 1:** Values of the specific heat capacity of graphite, $c$, as a function of temperature $T$ in Kelvin, as well as the correction factors determined for the NPL PSPC for a field of 12 × 5 cm^2^ at a water-equivalent depth of 5.2 g cm^−2^.

Parameter	Value
$c$ (J kg^−1^ K^−1^)	651.57 + 2.74 ($T$ − 273.15)
${k}_{\mathrm{imp}}$	1.0016
${k}_{\mathrm{gap}}$	1.0029
${s}_{\mathrm{w},\mathrm{g}}$	1.1210
${k}_{\mathrm{fl}}$	0.9713
${k}_{\mathrm{z},\mathrm{ cal}}$	1.0000

The sources of uncertainty for determining absorbed dose-to-water measured at CCHMC for a FLASH irradiation are presented in Table 2. The overall uncertainty accounts for uncertainties on the physical dimensions of the calorimeter components (used in the modelling), electrical calibrations of the thermistors, measurement of the specific heat capacity, beam-dependent Monte Carlo derived corrections, positioning, presence of a printed circuit board (PCB) within the calorimeter as well as uncertainties on the calorimeter measurements. The uncertainty budget for the NPL PSPC is dominated by uncertainties related to the dose conversion (0.71%), measurement of the specific heat capacity (0.26%) and the presence of the PCB (0.25%). The latter connects the thermistors to the instrumentation used to measure the calorimeter output. The ${s}_{\mathrm{w},\mathrm{g}}$ is strongly dependent on the mean excitation energies ($I$-values) used for water and graphite. In this work, the most recent recommended $I$-values for water (${I}_{\mathrm{w}}$ = 78 eV) and graphite (${I}_{\mathrm{g}}$ = 81 eV) were used^35^ and the associated uncertainty was determined based on a Monte Carlo sensitivity analysis carried out with various $I$-values chosen to be within their reported uncertainty. The ${k}_{\mathrm{fl}}$ factor is strongly dependent on the nuclear interaction cross-sections and the associated uncertainty was derived from the deviations found between the two Monte Carlo codes considered. The specific heat capacity was measured experimentally at NPL using a sample of graphite from the same block used to manufacture the calorimeter and the associated uncertainty was estimated from the experimental apparatus^36^. The PCB is positioned radially around the central axis of the core which could influence the measured signal. A series of tests were carried out to assess any possible perturbations and an associated uncertainty is included based on those measurements. The overall uncertainty is 0.9% at the 68% confidence level (1$\sigma$). This is in line with recommendations for absorbed dose measurements which should be performed with an uncertainty of the order of 1% (1$\sigma$)^37,38^. The latter was recommended based on clinical data for various types of tumours and indicates the level of uncertainty that should be achieved to ensure the optimal probability of eradicating the primary tumour.

**Table 2:** Relative uncertainty of absorbed dose-to-water measured by the NPL PSPC at CCHMC.

Sources of uncertainties (%)	Type A	Type B
Physical dimensions	< 0.01	0.09
Electrical calibrations	0.20	0.06
Specific heat capacity	0.08	0.26
${k}_{\mathrm{imp}}.{k}_{\mathrm{gap}}$	0.01	0.14
${s}_{\mathrm{w},\mathrm{g}}$. ${k}_{\mathrm{fl}}$	0.07	0.71
${k}_{\mathrm{z},\mathrm{ cal}}$	–	0.10
Positioning on beam axis and reference depth	0.06	0.13
PCB	–	0.25
Calorimeter measurements and analysis	0.04	0.15
Total	0.24	0.84
Overall (1$\sigma$)	0.9	

The average measured absorbed dose for the six fields by the NPL PSPC was 7.8 Gy with a standard deviation of 0.5%.

### Comparison with dose from ionisation chambers

**Figure 3:**
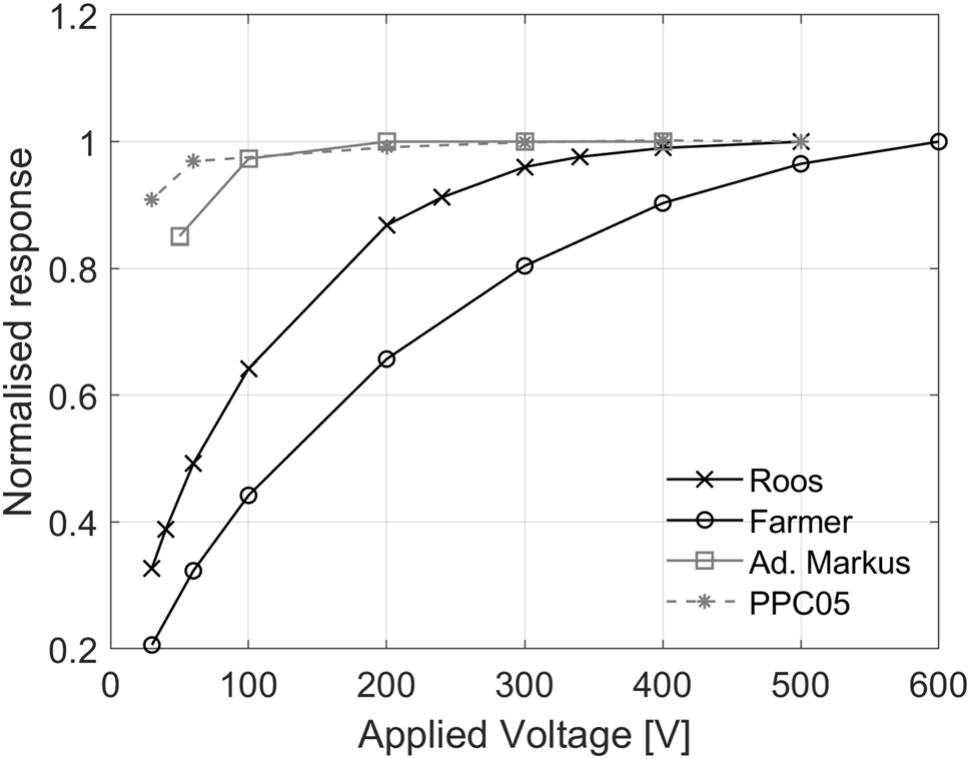
Normalised saturation curves measured for various ionisation chamber types. Data were normalised to the averaged measured signal for the maximum applied voltage for each ionisation chamber type. Note that the PTW Farmer operated at 600 V has not reached saturation.

Saturation curves measured for each ionisation chamber type are presented in Fig. 3. The efficiency of charge collection depends on the strength of the electrical field and consequently on the applied voltage. As the applied voltage increases, the collection efficiency, or saturation, increases. In theory, the saturated signal could be reached if a very large voltage is applied to the ionisation chamber, which in practice is not possible because of electrical breakdown of insulators or charge multiplication effects, where ions accelerated by the electrical field gain sufficient energy to further ionise the gas. On the other hand, at lower polarising voltages, a fraction of the positive and negative ions can recombine and a correction for lack of saturation—ion recombination—needs to be applied. According to established models for conventional radiotherapy, such as the Boag model^39^, the ion recombination should be inferred from lower polarising voltages where the collection efficiency is close to saturation. Table 3 presents the correction factors applied to each ionisation chamber type for an applied voltage of 400 V (collecting negative charge). The correction factor for ion recombination, ${k}_{\mathrm{ion}}$, was close to unity for the PTW Advanced Markus and IBA PPC05 ionisation chambers, independently of the method of measurement used, two-voltage^17,39^ versus three-voltage^40^**,** because at the operating voltage (400 V) the collection efficiency is close to saturation. These ionisation chambers have small sensitive volumes (0.02–0.05 cm^3^) and electrode spacing (0.6–1 mm) resulting in an increased strength of the induced electric field, hence collecting charge more effectively with reduced ion recombination in comparison with the PTW Roos and PTW Farmer (with sensitive volumes of 0.35 cm^3^ and 0.6 cm^3^, respectively). It is important to note that, plane-parallel ionisation chambers are commonly operated at lower voltages, in the order of 100 V, and it is common practice to operate them at larger voltages at high-dose rate beams to reduce ion recombination corrections. However, in small-electrode-spacing plane-parallel ionisation chambers (such as the PTW Advanced Markus and IBA PPC05), charge multiplication effects occur^41^ when operated at higher voltages. For the PTW Roos, the ion recombination correction varied between 4% and 5% and for the PTW Farmer between 14% and 21% depending on the method used. The two-voltage method is not accurate for ion recombination corrections larger than 3% because it relies on the linearity condition at lower polarising voltages where the collection efficiency is close to saturation, as described above, which does not hold for the PTW Roos and PTW Farmer ionisation chambers at an averaged dose rate of 63 Gy s^−1^ (Fig. 3). In summary, ion recombination corrections were derived using the three-voltage method for the dose determination from all ionisation chambers. The polarity, ${k}_{\mathrm{polarity}}$, correction factor was close to unity for all ionisation chambers except for the PTW Farmer, which can be traced to the large uncertainties on the collection efficiency at the operating voltage (400 V).

**Table 3:** Correction factors for ionisation chamber types at an averaged dose rate of ~ 63 Gy s^−1^ for an applied voltage of 400 V (collecting negative charge).

Ionisation chambers	${k}_{\mathrm{ion}}$, two-voltage	${k}_{\mathrm{ion}}$, three-voltage	${k}_{\mathrm{polarity}}$
PTW-34001 Roos	1.046	1.039	1.002
PTW-30013 Farmer	1.143	1.212	0.968
PTW-34045 Ad. Markus	1.000	1.000	1.001
IBA PPC05	1.003	1.001	1.000

**Figure 4:**
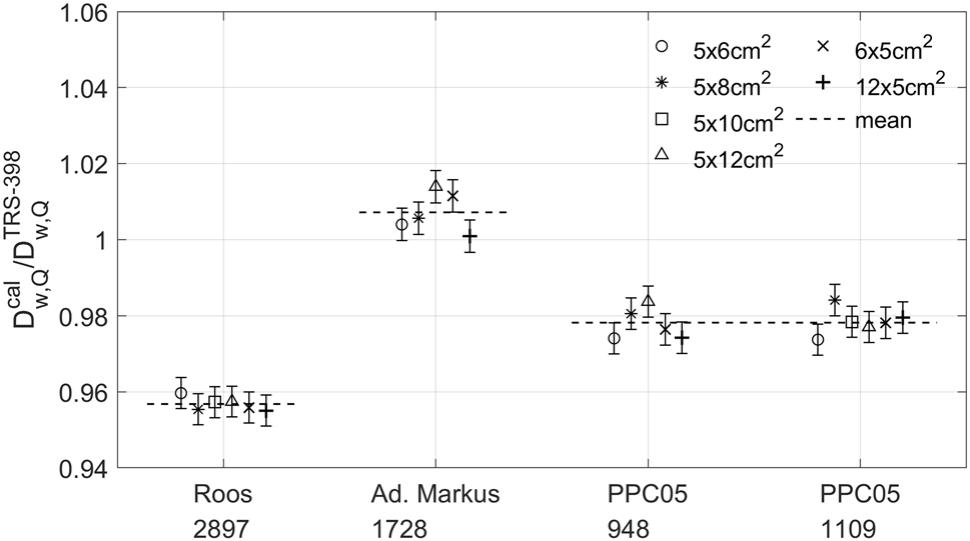
Ratio between the dose determined by the NPL PSPC and the dose derived from ionisation chambers for the various fields tested. The dashed lines represent the mean ratio value for each ionisation chamber type and the error bars represent type A standard uncertainties.

Figure 4 shows the ratio between the dose determined by the NPL PSPC and the dose derived from ionisation chambers for the six fields tested. The PTW Farmer was not considered in the comparison due to the large uncertainties on the determination of ion recombination corrections as well as the limited allocated experimental time. There was a consistent difference between the dose determined by the NPL PSPC and the dose derived from ionisation chambers for each ionisation chamber type. The better agreement was found for the PTW Advanced Markus (1.007) and IBA PPC05 (0.978), whereas larger deviations were found for the PTW Roos (0.957), the latter indicating an overestimation on the ion recombination correction applied. Considering an uncertainty of 2.3% (1$\sigma$)^17^ for the dose derived from ionisation chambers, the PTW Advanced Markus and IBA PPC05 chambers agreed within uncertainties with the dose determined from the NPL PSPC. Note that this quoted uncertainty is for ionisation chamber measurements performed at conventional dose-rates, therefore ion recombination uncertainties are underestimated. Nevertheless, small-electrode-spacing ionisation chambers operated at higher voltages can be used for reference dosimetry of FLASH proton beams at averaged dose rate of 63 Gy s^−1^. Despite the larger deviation for the dose measurements between the PTW Roos and the NPL PSPC, the dose measurements for this chamber showed very good repeatability. Therefore, this chamber could be used in these beams for reference dosimetry if the ion recombination correction is inferred from the comparison with the absorbed dose determined with the NPL PSPC^24^.

## Conclusion

Calorimetry measurements were performed, and necessary correction factors established for absolute dosimetry of FLASH proton pencil beam scanning. This enabled the safe and accurate implementation in the clinic of this new treatment modality. The NPL PSPC accurately measures the dose delivered with an uncertainty two times smaller than the dose derived from ionisation chambers. The response of the calorimeter is dose-rate independent, as opposed to the response of ionisation chambers which need to be very well characterised at FLASH dose-rates since large ion recombination effects occur. The overall uncertainty on the dose measured with the NPL PSPC is 0.9% (1$\sigma$) which is in line with recommendations^37,38^ for reference dosimetry for effective radiotherapy treatments.

Although absolute dosimeters such as calorimeters are very accurate, they are complex and not suitable for routine clinical measurements. Therefore, calorimetry measurements were compared against measurements performed with ionisation chambers, which are simpler to operate, to assess the feasibility of using these detectors in UHDR proton beams for reference dosimetry and quality assurance of treatments. Plane-parallel ionisation chambers with small electrode spacing showed good agreement with the NPL PSPC and thus can be used for reference dosimetry as well as for quality assurance of FLASH proton pencil beam scanning treatments delivered at an averaged dose rate of ~ 63 Gy s^−1^. The establishment of a calorimeter for absolute dose measurements of proton FLASH irradiations improves accuracy and consistency, ensuring optimal dose delivery for this new treatment modality. This provides assurance for the implementation of FLASH proton beam radiotherapy into clinical practice. However, it is worth noting that for other FLASH radiotherapy modalities, such as high-energy electron^21^ and very high-energy electron beams^42^, ionisation chambers suffer from much larger ion collection losses^21,24^. This is due to significantly different temporal beam structure as compared to FLASH proton pencil beams used in this work. These ion collection losses, which can be as large as 90%^24^, are heavily dependent on the dose-per-pulse and cannot be predicted by any of the existing analytical ion recombination models. In these circumstances, calorimetric methods are a superior solution in determination of the absorbed dose.

In summary, this work demonstrates the establishment of a calorimeter for absolute dose measurements of proton FLASH irradiations. The method presented improves accuracy and consistency of the dose delivered and is crucial for the safe implementation of clinical trials, and beyond, for this new treatment modality.

## Methods

### Measurements

The experiments have been conducted at the CCHMC beamline, equipped with a 250 MeV proton cyclotron (ProBeam, Varian Medical Systems, Palo Alto, CA, USA). The CCHMC facility has a research-dedicated gantry room which has been upgraded to deliver FLASH irradiations. In advance of a feasibility trial named FAST-01^16^ for the treatment of symptomatic bone metastasis, six rectangular vertical fields of 5 × 6, 5 × 8, 5 × 10, 5 × 12, 6 × 5 and 12 × 5 cm^2^ were tested using a transmission 250 MeV mono-energetic scanned layer at an averaged dose rate of ~ 63 Gy s^−1^, defined according to^32^. In a transmission field, the plateau region of a mono-energetic proton beam is used for treatment. All fields were tuned to deliver a planned physical dose of ~ 8 Gy in one fraction for the FAST-01 trial. The time of delivery ranged between 400 and 800 ms, depending on the field size. The FLASH effect is likely dependent on a combination of multiple parameters (e.g. irradiation type, delivered dose, endpoint), and, in terms of delivery time, it has been reported for a wider range of delivery times^42,43^. To verify and validate the planned dose independently, absolute dose measurements were performed using the NPL PSPC.

**Figure 5:**
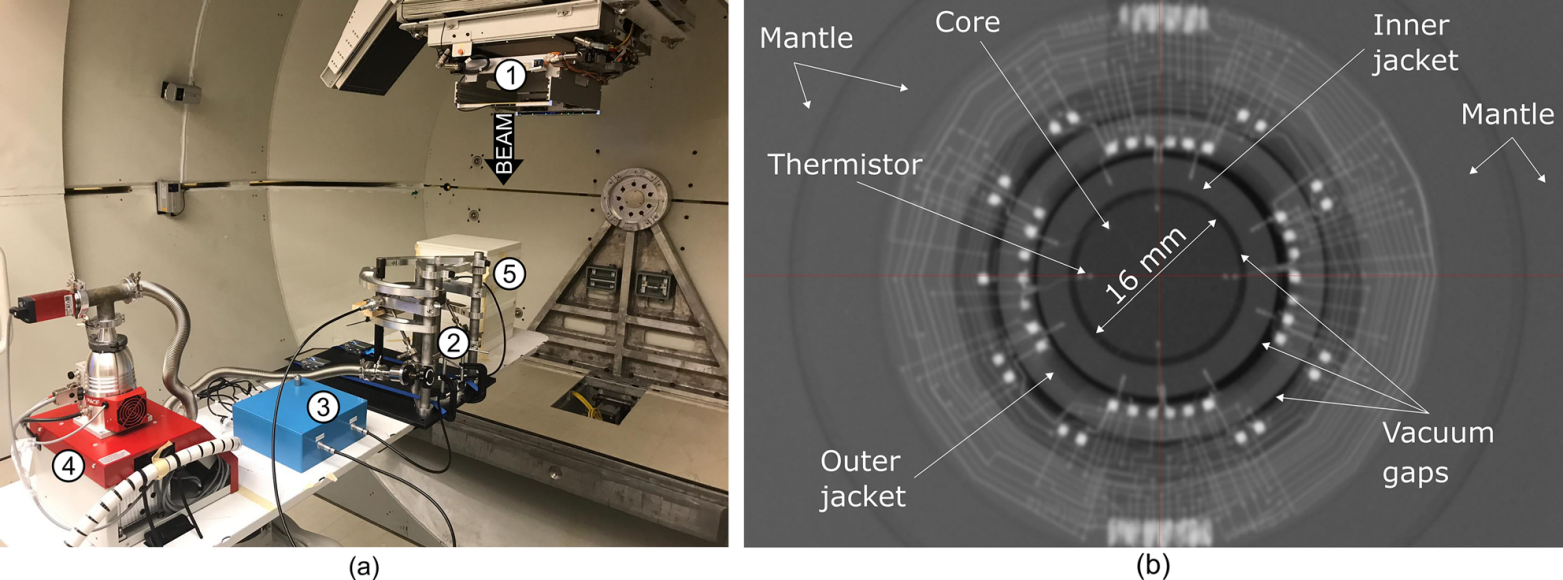
Experimental setup mounted on the patient couch and radiograph of the NPL PSPC. (**a**) (1) Gantry, (2) NPL PSPC, (3) measurement instrumentation for the NPL PSPC, (4) vacuum pump, (5) ionisation chamber setup. (**b**) Radiograph of the front view of the NPL PSPC^44^.

Measurements were performed at a water-equivalent thickness of 5.2 g cm^−2^ using the NPL PSPC and ionisation chambers (Fig. 5a). The reference point of the calorimeter and ionisation chambers was positioned at the isocentre on the central axis of the vertical beam, and the source-to-detector distance (SDD) was kept constant to avoid corrections for the divergence of the beam. Accurate positioning was achieved using the room laser-alignment system and the electronically controlled 6-axis patient couch. Calorimeter and ionisation chamber measurements were carried out on the same day to avoid day-to-day variations of the beam output.

### NPL PSPC system and correction factors

The calorimeter consists of disc shaped graphite components that are arranged in a nested configuration (Fig. 5b). The core, 2 mm thick and 16 mm diameter, is enclosed within two sets of jackets (inner and outer) and a mantle. The jackets and mantle are manufactured in two halves, consisting of front and back components and the volume within the mantle is maintained in a high-quality vacuum to minimise the heat-transfer between components and the environment. Around the circumference of each component, small-bead, hermetically sealed thermistors, type GE Sensing GC7300, are equidistantly embedded at a depth of approximately 2 mm below the surface of each component. The core contains four thermistors, two of which are used for temperature sensing and two for heating the graphite; the inner and the outer jacket halves each contain eight thermistors, four of which are used as temperature sensors and four as heaters; the mantle lid and the mantle base each contain two temperature-sensing thermistors. A radial shaped PCB is positioned between the outer front and back jackets to manage the thermistor wiring for the components located within the calorimeter. The wires then pass via vacuum feed-through connectors onto an interface box positioned close to the calorimeter but outside the area of the direct radiation beam (Fig. 5a). The temperature sensing thermistors in each component are electrically connected into separate networks each of which is connected to its own direct current (DC) Wheatstone bridge. The core, however, has two independent temperature sensing thermistors, each one connected to its own DC Wheatstone bridge circuit. Bridge out-of-balance voltage data from each DC Wheatstone bridge circuit are acquired by individual Keithley K2182A nanovoltmeters. In total, eight temperature sensing networks are connected to eight DC Wheatstone bridges and K2182A nanovoltmeters (two in the core, two in the inner-jacket—front and back, two in the outer-jacket—front and back, and two in the mantle—lid and base). A cable of approximately 25 m length connects the interface box located close to the calorimeter to the instrumentation and DC Wheatstone bridges located in the control room. The thermistors that are used to heat each of the graphite components are also electrically connected into networks. The two heating thermistors in the core form one network, whilst the thermistors located in the inner-front, inner-back, outer-front and outer-back jackets, are connected into one network for each component resulting in a total of five heating networks. Both the sensor and heating networks consist of thermistors with almost identical resistance connected in parallel-series networks, which have approximately the same resistance as the individual thermistors. By distributing the thermistors of each network evenly over the perimeter of the graphite component, the average temperature of the component is measured with a single bridge or the heat from a single power source is dissipated more evenly within the component, thus reducing thermal gradients and minimising measurement errors. A National Instruments (NI) PXI-1045 chassis is loaded with ten NI PXI-4070 digital multimeters (DMMs), five NI PXI-4110 power supply units (PSUs), and one NI PXI-6602 counter-timer. The counter-timer provides a hardware timed clock pulse to drive the external triggers of the ten NI PXI-4070 DMMs and eight Keithley K2182A nanovoltmeters. The PXI-4110 PSUs provide electrical power to each of the thermistor heater networks whilst the PXI-4070 DMMs monitor the voltage drop across each heating network and the voltage drop across a precision resistor which is electrically positioned in series with each network to enable current in the heater circuits to be calculated. The product of thermistor network voltage and current allows the electrical energy dissipated in the heater networks to be calculated. The instrumentation is controlled by a computer running a LabVIEW v2010 control and data acquisition program, and the data is analysed off-line using an in-house developed MATLAB R2016b program.

In this work, the calorimeter was operated in quasi-adiabatic mode. This mode maintains the outer jacket at a constant temperature (using its heating thermistors), a few degrees above the irradiation room temperature, whilst the temperature in the core and inner jacket are allowed to drift. This provides a more stable environment to the core as it suppresses any temperature changes in the treatment room. Note that the calorimeter can also be operated in active isothermal mode^31^. However, this was not possible in the allocated experimental time. In this mode, all components are always maintained at a constant temperature, including throughout irradiation. Unlike measurements with longer irradiation times where a difference in dose was found between the two modes of operation due to uncertainties on heat transfers between the calorimeter components^31^, here we expect the difference to be negligible given the fast delivery of UHDR beams. To position the calorimeter core at the correct water-equivalent depth, graphite build-up plates were placed in front of the calorimeter. For a reference depth in water of 5.2 g cm^−2^, the equivalent graphite mass thickness is 5.9 g cm^−2^^45^. Data were collected at five samples per second and irradiations were repeated at least twenty times for each field tested to achieve a relative standard uncertainty of 0.04% (Type A). Irradiations were repeated every 2 min. For each irradiation, a quadratic fit as a function of time is applied to the pre- and post- irradiation drift curves. These drifts are extrapolated and the increase in temperature is determined from the offset between the two drifts (Fig. 1). The average absorbed dose to the core is determined by multiplying the measured increase in temperature by the specific heat capacity of the core, which was determined experimentally at NPL^36^. The absorbed dose-to-core is converted to absorbed dose-to-water by the product of the necessary beam-dependent correction factors which were determined using Monte Carlo simulations. (1) The impurity correction factor, ${k}_{\mathrm{imp}}$, accounts for the presence of non-graphite components in the calorimeter geometry, such as thermistors, epoxy resin (to hold the thermistors), and thermistor wires. As fractions of the total mass of the core, the thermistors comprise 0.48%, the epoxy resin 0.23% and the wires 0.13%. ${k}_{\mathrm{imp}}$ is determined as the ratio between the dose averaged over the core in a pure graphite geometry (with vacuum gaps) and the dose averaged over the core in the real geometry with all the non-graphite components. (2) The gap correction, ${k}_{\mathrm{gap}}$, accounts for the presence of vacuum gaps within the calorimeter to minimise heat transfers. ${k}_{\mathrm{gap}}$ was determined by modelling the calorimeter with a compensated geometry. This compensated geometry entailed shifting the calorimeter jackets in the simulation to remove the vacuum gaps, whilst maintaining the same depth of graphite in front of the core for it to be at the correct water-equivalent thickness and filling up the vacuum spaces with graphite in the simulation. The ratio of the averaged dose over the core in the compensated geometry, with no vacuum gaps, relative to the averaged dose over the core constituting of pure graphite with vacuum gaps (described alongside ${k}_{\mathrm{imp}}$ determination method), gives ${k}_{\mathrm{gap}}$ ^46^. The product ${k}_{\mathrm{imp}}$. ${k}_{\mathrm{gap}}$ converts the dose-to-core in the real calorimeter geometry to dose-to-graphite in a geometry composed of homogeneous graphite. Dose-to graphite is converted to dose-to-water by (3) the water-to-graphite mass-stopping-power ratio, ${s}_{\mathrm{w},\mathrm{g}}$, and (4) the fluence correction factor, ${k}_{\mathrm{fl}}$, to correct for the difference in fluence at water-equivalent depths between water and graphite. The ${s}_{\mathrm{w},\mathrm{g}}$ ratio is determined as an integral over the charged particle spectrum and ${k}_{\mathrm{fl}}$ determined from the charged particle fluence differential in energy between water and graphite^33^.

### Monte Carlo simulations

To determine the correction factors, accurate values of mass of all calorimeter components, their physical dimensions, and properties as well as accurate modelling of the beam parameters and experimental apparatus were considered in the simulations. Monte Carlo simulations were performed with TOPAS code v3.6.1 based on Geant4 v10.6.p03^47^ and FLUKA-2021.2.0 code^48,49^. Materials were defined according to recommendations of the International Commission on Radiation Units and Measurements Report 90^35^. Simulations with TOPAS were carried out using the default modular physics list which includes the reference hadronic physics list g4h-phy_QGSP_BIC_HP as well as the standard electromagnetic physics models (g4em-emstandard_opt4). FLUKA simulations were performed using the default card HADROTHErapy. The use of two different Monte Carlo codes gives confidence on the Monte Carlo beam dependent corrections derived for the calorimeter. Unlike ${k}_{\mathrm{imp}}$, ${k}_{\mathrm{gap}}$ and ${s}_{\mathrm{w},\mathrm{g}}$, ${k}_{\mathrm{fl}}$ varies significantly depending on the nuclear models implemented in a given Monte Carlo code and, at present, estimates for nuclear data uncertainties are not provided. The ${k}_{\mathrm{fl}}$ factor was determined experimentally and from Monte Carlo simulations in previous work^45^. Although, ${k}_{\mathrm{fl}}$ determined by experiments was found to represent a partial fluence correction, good agreement was found between partial fluence corrections determined experimentally and those calculated by FLUKA when the experimental setup was simulated. For this reason, ${k}_{\mathrm{fl}}$ determined by FLUKA was considered and the use of two different Monte Carlo codes allowed an estimate of its uncertainty. The uncertainty was determined based on an asymmetric triangular distribution. The limits of the triangular distribution were chosen considering the difference found on the estimation of ${k}_{\mathrm{fl}}$ between the two codes. To benchmark the simulated beam parameters, an IDD curve was measured with a large-area ionisation chamber (IBA StingRay, radius of 12 cm) at conventional dose rate as well as lateral profile measurements carried out at the reference depth with EBT3 film for the six fields tested at a dose rate of 63 Gy s^−1^ for a 250 MeV proton beam. These experimental data were used to optimise the simulated beam energy, Gaussian energy spread and beam divergence iteratively and it was assumed that these parameters do not change between conventional and FLASH dose rates. Optimisation consisted of obtaining the minimal difference between the simulated and measured data for beam range and Bragg peak width (better than 0.1 mm), the IDD’s point-to-point dose differences (within 2%) and the full-width at half-maximum (FWHM) of the lateral profiles (better than 0.1 mm).

### Films

Gafchromic® film (EBT3, Ashland, NJ) was calibrated at the clinical proton pencil beam scanning facility at The Christie, UK, using a 245 MeV beam. The calibration EBT3 films were exposed to a 10 × 10 cm^2^ field size at a depth of 2 g cm^−2^ in a water-equivalent plastic phantom made of RW3 (PTW, Freiburg, Germany). Twelve pieces of EBT3 film of approximately 3.5 × 3.5 cm^2^ have been irradiated in equal absorbed dose steps ranging from 0 to approximately 10 Gy. The number of EBT3 film pieces and dose levels were chosen from recommended values by previous work^50^. Subsequently, EBT3 films were subjected to the 250 MeV FLASH proton beam at the CCHMC in a RW3 phantom (IBA-Dosimetry, Schwarzenbruck, Germany) at the required water-equivalent depth of 5.2 g cm^−2^ for all the six field sizes in consideration. EBT3 films have been digitized in a 48-bit RGB signal with 150 dpi spatial resolution using an Epson Expression 10000XL flatbed scanner operating in the transmission mode without colour corrections. The film characterisation protocol used in this work has been previously descried^51^. The Gafgui version 4.0^50,52^ software was employed to analyse the EBT3 films using the methods described in^51^.

### Ionisation chambers

Measurements were performed using four plane-parallel ionisation chambers (PTW 34001 Roos #2897, PTW Advanced Markus 34045 #1728, two IBA PPC05 #948 and #1109) and one cylindrical ionisation chamber (PTW 30013 Farmer #10507). The PTW Advanced Markus #1728 and IBA PPC05 #948 are ionisation chambers used clinically at CCHMC and were previously calibrated in terms of absorbed dose to water in a ^60^Co beam^17,18^ at the National Institute of Standards and Technology (NIST, US), whilst the PTW Roos #2897, the IBA PPC05 #1109 and the PTW Farmer #10507 were calibrated at NPL (UK). Ionisation chamber measurements were performed in a phantom consisting of water-equivalent plastic material (Fig. 5a). For convenience of the experimental setup the CCHMC’s ionisation chamber measurements were performed using a water-equivalent plastic phantom made of high-density polyethylene (HDPE), and for the other chambers RW3 (IBA-Dosimetry, Schwarzenbruck, Germany) was used. Both materials scale in a similar way to water and 5 cm of plastic material were used in front of the chambers to achieve the required water-equivalent depth of 5.2 g cm^−2^. A material-dependent fluence scaling factor should be used^17^ to correct for the difference in the ionisation chamber readings in water and the solid-state water-equivalent phantoms. This correction increases with depth and since measurements were performed at a shallow depth (5.2 g cm^−2^) in comparison with the beam range (38 g cm^−2^), it can be assumed that this correction factor is close to unity^53^. For the plane-parallel ionisation chambers, the reference point was taken to be 1 mm deep inside the chamber front face, whereas for the cylindrical ionisation chamber, it was taken to be at the ionisation chamber central axis.

All ionisation chambers were operated with an applied voltage of − 400 V (collecting negative charge, the collecting electrode was positive with respect to the polarising electrode). The absorbed dose is determined by multiplying the reading of the ionisation chambers (corrected to standard temperature, 293.2 K for NPL calibrations and 295.2 K for NIST calibrations, and pressure, 1013.3 mBar, electrometer corrections, polarity and ion recombination) by the calibration coefficients provided by the standards labs (from NIST and NPL, respectively) and a beam quality correction factor, ${k}_{Q,{Q}_{0}}$, to account for the difference between the ionisation chamber response in ^60^Co (the beam used to calibrate them) and that in a proton beam (the user beam). For the PTW Roos and PTW Farmer chambers, the ${k}_{Q,{Q}_{0}}$ values recommended by the International Atomic Energy Association Technical Report Series #398^17^ (IAEA TRS-398) were used. As IAEA TRS-398 does not report ${k}_{Q,{Q}_{0}}$ values for the PTW Advanced Markus and IBA PPC05, the reported values from the proceedings of the 25th Council on Ionizing Radiation Measurements and Standards^54^ were applied.

Saturation curves for all ionisation chambers tested were measured to demonstrate the chamber response as a function of the applied voltage. Regarding ion recombination corrections, the dependence of the reciprocal of the ionisation chamber reading on the reciprocal of the applied polarising voltage (Jaffé plot) was first assessed to ascertain whether the beam should be regarded as pulsed (linear) or continuous (quadratic polynomial) in relation to ion recombination. The results showed a quadratic relation, hence the quadratic expression for the two-voltage method^17^ was used to determine ion recombination corrections. Ion recombination corrections were also determined using the three-voltage method^40^ with a fit at higher voltages. Although charge multiplication occurs at higher voltages^41^, the very low efficiency charge collection at lower voltages (Fig. 3) for some ionisation chamber types tested in UHDR showed that a fit at higher voltages provides a more accurate estimate of the ion recombination correction.

## Data Availability

The datasets generated during and/or analysed during the current study are available from the corresponding author on reasonable request.
